# Injury Pattern and Current Early Clinical Care of Pediatric Polytrauma Comparing Different Age Groups in a Level I Trauma Center

**DOI:** 10.3390/jcm13020639

**Published:** 2024-01-22

**Authors:** Anna Schuster, Lisa Klute, Maximilian Kerschbaum, Jürgen Kunkel, Jan Schaible, Josina Straub, Johannes Weber, Volker Alt, Daniel Popp

**Affiliations:** 1 Department of Trauma Surgery, University Medical Centre Regensburg, 93053 Regensburg, Germanyjosina.straub@ukr.de (J.S.); johannes1.weber@ukr.de (J.W.); volker.alt@ukr.de (V.A.); 2 Department of Pediatrics, KUNO University Children’s Hospital Regensburg, 93053 Regensburg, Germany; 3 Conradia Radiologie München, 80798 München, Germany; jan.schaible@ukr.de

**Keywords:** severely injured, children, pediatric trauma, polytrauma, injury patterns, mortality

## Abstract

**Introduction:** Pediatric polytrauma is a complex condition with unique characteristics and requirements for early clinical care. This study aimed to analyze the injury patterns, early clinical care, and outcomes of pediatric polytrauma patients in a Level I trauma center. The focus was on evaluation between different age groups and the recognition of injuries as potential factors influencing outcomes. **Methods:** A prospective cohort study model of pediatric polytrauma patients (ISS ≥ 16) was conducted over a 13-year period, stratified by age groups (Group A: 0–5 years; Group B: 6–10 years; Group C: 11–15 years; and Group D: 16–18 years). A comparison of the groups was conducted to examine variations in early clinical care, trauma mechanisms, distribution of affected body regions (as per AIS and ISS criteria), and trauma-related mortality. Additionally, factors contributing to mortality were evaluated. **Results:** The median age of patients was 16 years, with a male predominance (64.7%). The Injury Severity Score (ISS) varied across age groups, with no significant difference. The 30-day mortality rate was 19.0%, with no significant age-related differences. Trauma mechanisms varied across age groups, with motor vehicle accidents being the most common mechanism in all age groups except 0–5 years, where falls were prevalent. Analysis of injury patterns by AIS body regions indicated that head trauma was a significant predictor of mortality (Hazard Ratio 2.894, *p* < 0.001), while chest, abdominal, and extremity trauma showed no significant association with mortality. Multiple regression analysis identified the ISS and preclinical GCS as valid predictors of mortality (*p* < 0.001 and *p* = 0.006, respectively). **Conclusions:** While age-related differences in injury severity and clinical interventions were limited, head trauma emerged as a critical predictor of mortality. Early recognition and management of head injuries are crucial in improving outcomes. Additionally, the ISS and preclinical GCS were identified as valid predictors of mortality, emphasizing the importance of early assessment and resuscitation. A tailored approach to pediatric polytrauma care, considering both age and injury patterns, might contribute to survival benefits in this vulnerable population.

## 1. Introduction

Polytrauma in the pediatric population is a relatively rare occurrence, accounting for just 7.4% of all cases in the German Trauma Registry from 1997 to 2010 [1]. This translates to about 270 cases of pediatric polytrauma annually in Germany. The proportion of those under 15 years old is even smaller, at approximately 3% [2]. Nevertheless, trauma in childhood remains the most common cause of hospital admissions and stands as the predominant cause of death in children beyond their first year of life [3,4,5]. 

Polytraumatic injuries in children and adolescents pose a unique challenge for healthcare providers. To ensure a well-established and tested workflow, the S2k guideline “Polytrauma Management in Childhood” recommends aligning the shock room management with the concept developed for adults, known as “Advanced Trauma Life Support” [5]. Rapid recognition of critical conditions and prompt intervention are crucial in managing these cases to achieve optimal outcomes. To ensure effective care, it is essential to understand the injury patterns while considering anatomical and age-specific characteristics in the pediatric population. Both the diagnostic process and the choice between surgical and non-surgical approaches depend on the nature and distribution of injuries. While numerous studies of errors in the treatment of severely injured patients have been conducted in adults [6,7,8], only a paucity of such studies have been performed concerning children and adolescents. In general, children with polytrauma should be increasingly treated at regional trauma centers [1], since specialized trauma centers are associated with lower mortality rates, improved survival in severe head injuries, and reduced organ loss in blunt abdominal trauma [9,10,11].

Through this work, we aim to gain new insights into the interdisciplinary care of polytraumatized children and adolescents in a German Level I trauma center. This study addresses two relevant questions, which will be answered through the prospective investigation of severely injured patients:

- Are there significant differences in injury patterns, early clinical care, and treatment outcomes among different age groups within the pediatric population of severely injured patients?
- Which injured body regions are associated with higher mortality rates in severely injured pediatric patients (ISS > 16)?

By addressing these questions, we aim to deepen our understanding of pediatric polytrauma and ultimately improve the care and outcomes of this vulnerable patient group.

## 2. Materials and Methods

### 2.1. Study Design and Inclusion Criteria

A prospective cohort study was chosen to investigate relevant differences between age groups regarding injury pattern, early clinical care, and factors correlated with the mortality of pediatric polytrauma patients. Patients admitted through our emergency department (Level 1 trauma) between 2007 and 2019 were recruited for this study. Inclusion criteria were an Injury Severity Score (ISS) of ≥16 and an age at admission of ≤18 years. Cases not meeting the above-mentioned criteria were excluded. Additionally, cases with inadequate data documentation were excluded. Gender-based exclusion of patients was not applied. The collection of data was performed by study assistants around the clock, seven days a week.

The included patients were divided into four groups and compared. The grouping was based on age (Group A: 0–5 years; Group B: 6–10 years; Group C: 11–15 years; Group D: 16–18 years). Age, gender, Injury Severity Score (ISS) as described by Baker et al. [12], prehospital medical care, clinical interventions, length of hospital stay, injury pattern, trauma mechanisms, and 30-day mortality rate were recorded. Injury severity overall was assessed using the Abbreviated Injury Score (AIS), with categories ranging from 1 (minor) to 6 (maximum, currently untreatable). These categories include 2 (moderate), 3 (severe, not life-threatening), 4 (serious, life-threatening), 5 (critical, survival uncertain), and 6 (maximum, currently untreatable). Comparisons were made among groups based on the ISS in different body regions, including the head, chest, abdomen/pelvis, and extremities. The choice of the ISS over alternative scales was due to its outstanding and widespread role in evaluating injury severity, coupled with its prevalence in national trauma registers and will be extensively discussed in the forthcoming Discussion section. Calculated from the squared Abbreviated Injury Scale (AIS) severity scores of the three most severely affected body regions, the ISS provides a comprehensive measure. For instance, if a patient has an AIS score of 3 for head injuries, 4 for thoracic injuries, and 2 for abdominal injuries, the ISS would be = (42) + (32) + (22) = 16 + 9 + 4 = 29.

*ISS* = (*x*^2^) + (*x*^2^) + (*x*^2^)

### 2.2. Ethics Statement and Anonymity

This study received approval from the institutional ethical review board in accordance with the Declaration of Helsinki (14-101-0004). Patient anonymity was ensured. At the beginning of documentation, a multi-digit identification number was assigned to each individual case.

### 2.3. Statistical Analysis

Descriptive statistics were employed for nominal scale variables using absolute frequencies (*n*) and relative frequencies (%). Metric variables were assessed for normal distribution through histogram analysis, QQ plots, and the Kolmogorov–Smirnov test. As normal distribution was mostly not met within subgroups, they were consistently reported as median and 25th and 75th percentiles (MD (25th; 75th percentile)).

Differences between age groups for nominal scale variables were determined using cross-tabulations and the Chi-square test. In cases where expected cell frequencies were ≤5, Fisher’s exact test was computed as an alternative. Effect size was reported as Phi for 2 × 2 cross-tabulations and Cramer’s V for cross-tabulations with more than 2 × 2 cells. A Phi/V of 0.1 indicated a small effect, 0.3 a medium effect, and 0.5 a large effect. To test for differences between age groups regarding metric, non-normally distributed variables, and ordinal scale variables, the Kruskal–Wallis test was performed. If this test was significant, Mann–Whitney U tests were calculated as part of post-hoc analyses. Effect size was reported as r, where an r of 0.1 corresponded to a small effect, 0.3 to a medium effect, and 0.5 to a large effect. Correlations between nominal variables were examined using the symmetry measure Phi for 2 × 2 cross-tabulations and Cramer’s V for cross-tabulations with more than 2 categories.

To identify predictors of 30-day mortality, Cox regressions were conducted. Initially, univariate Cox regressions were computed for each predictor individually, reporting the Hazard Ratio (HR) and its corresponding 95% confidence interval [95% CI]. To account for potential multicollinearity among predictors, a multiple Cox regression was subsequently performed, including all predictors that became significant in the univariate analysis, using a stepwise backward algorithm with a threshold of *p* = 0.07. Within the final model, the Hazard Ratio and its corresponding 95% CI were reported for each predictor once again.

The statistical analysis (level of significance, *p* < 0.05) was carried out using SPSS software version 29 (SPSS Inc., Chicago, IL, USA).

## 3. Results

### 3.1. Demographic Data

In total, 184 patients met the inclusion criteria; demographic data are shown in Table 1. As the age group advanced, there was a corresponding rise in the percentage of male patients (*p* = 0.049). The Injury Severity Score (ISS) varied across age groups, with no significant difference. Prehospital Glasgow Coma Scale (GCS) scores and intubation rates were similar among age groups. The intubation rate for the entire patient population was 76.6%. In the youngest age group, 91.7% were intubated, while in the oldest age group, 71.6% were intubated. Thoracic drainage and cardiopulmonary resuscitation (CPR) rates did not differ significantly. Overall, 13.0% of patients were resuscitated in the preclinical setting. Among these, 79.2% experienced only temporary resuscitation success until arrival in the trauma bay. In the 0–5-year-old group, 20.8% were resuscitated, with 60.0% of these resuscitations being successful until admission to the trauma bay. In the 16–18-year-old group, 11.6% underwent resuscitation, with an 82.8% success rate. The 30-day mortality rate was 19.0%, with no significant age-related differences. Time of hospitalization also showed no significant variation across age groups. 

### 3.2. Mechanisms of Injury

**Figure 1:**
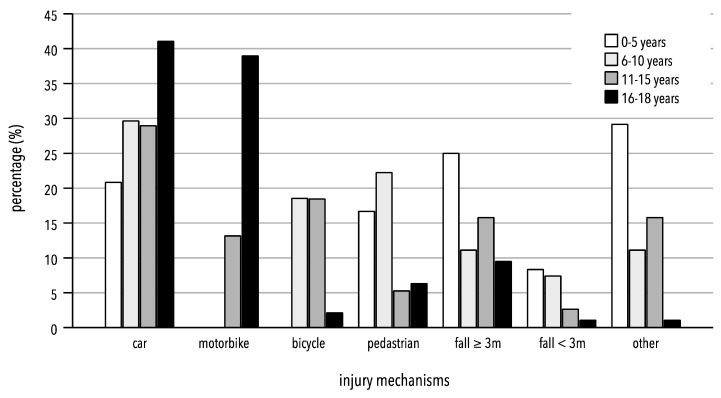
Percentage distribution of trauma mechanisms in different age groups. Others: suicide, violence, shaken baby syndrome, accident in agriculture.

Within the entire cohort, 34.2% of polytrauma cases were caused by car accidents (see Table 2). Although the proportion of car accidents increased with the age of the groups, this association was not statistically significant (*p* = 0.203). From the age group of 6–10 years onward, car accidents became the most common cause of polytrauma (see Figure 1). Motorcycle accidents accounted for 22.8% of the overall population. Motorcycle accidents were significantly more frequent in the 16–18-year-old group compared to all other groups (*p* < 0.001). In this age group (16–18 years), motorcycle accidents were the second-most common cause of injuries. The analysis also revealed that bicycle accidents were significantly more common in the 6–10 years and 11–15 years age groups (*p* < 0.001) compared to the other two age groups. Overall, 7.6% of the cohort were involved in bicycle accidents. In total, 9.8% of the cohort suffered injuries as pedestrians. There was a varying distribution across different age groups (*p* = 0.042), with the highest proportion (22.2%) in the 6–10-year-old age group. Falls from heights of ≥3 m accounted for 13.0% of the entire cohort’s polytrauma cases. No significant differences were observed among the various age groups for this mechanism of injury (*p* = 0.218). Similar findings were observed for falls from moderate heights (<3 m). Here, too, there were no differences between the age groups (*p* = 0.176). 

In the 0–5-year-old age group, other mechanisms of injury, such as accidents in agriculture or being kicked by a hoof, were most common (29.2%; see Figure 1). There was a significant variation in distribution among the age groups (*p* < 0.001)

### 3.3. Injury Pattern

Collectively, most injuries with an AIS score of ≥2 were primarily located in the head region across all age groups (distribution of injury patterns is shown in Table 3). Furthermore, this was the sole anatomical area where the two youngest age groups exhibited significantly higher rates of injury compared to their older counterparts (*p* = 0.002; refer to Table 4). In detail, 95.8% of patients in the youngest age group (0–5 years) and 92.6% in the second-youngest age group (6–10 years) experienced head injuries. For the entire study cohort, the median AIS score for head injuries was 4. Thoracic injuries affected 70% of the patients, with no statistically significant variation observed among different age groups (*p* = 0.700). Conversely, injuries to the abdomen were the least prevalent across all age categories. Notably, as the age of the patient groups increased, there was a corresponding rise in the proportion of abdominal injuries (*p* < 0.001). Similar trends were noted for severe limb injuries, with higher age groups exhibiting a greater incidence of these injuries (*p* = 0.004).

### 3.4. General Influence of Resuscitation on Mortality

**Figure 2:**
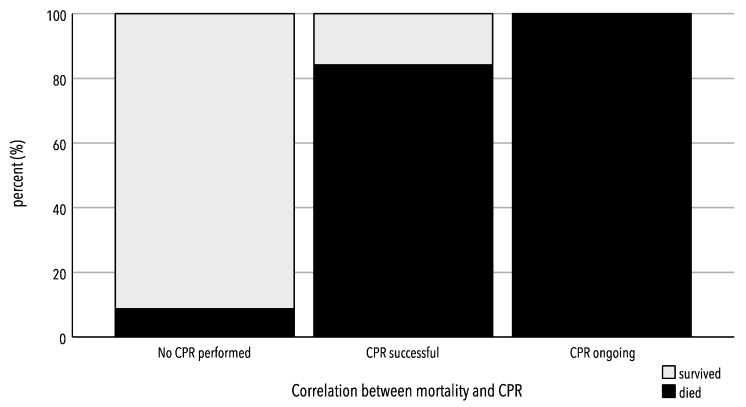
Correlation between CPR and mortality.

A significant impact of the resuscitation rate on mortality was observed (*p* < 0.001), as shown in Figure 2:

Patients who were successfully resuscitated before arriving at the hospital and patients who were admitted under resuscitation showed significantly higher mortality rates compared to the group of non-resuscitated patients (no resuscitation: 8.8% (*n* = 14); vs. short/successful resuscitation: 84.2% (*n* = 16)). No significant difference was observed in the mortality rate between patients with successful resuscitation and those with ongoing resuscitation.

### 3.5. Thirty-Day Survival/Mortality Rate

**Figure 3:**
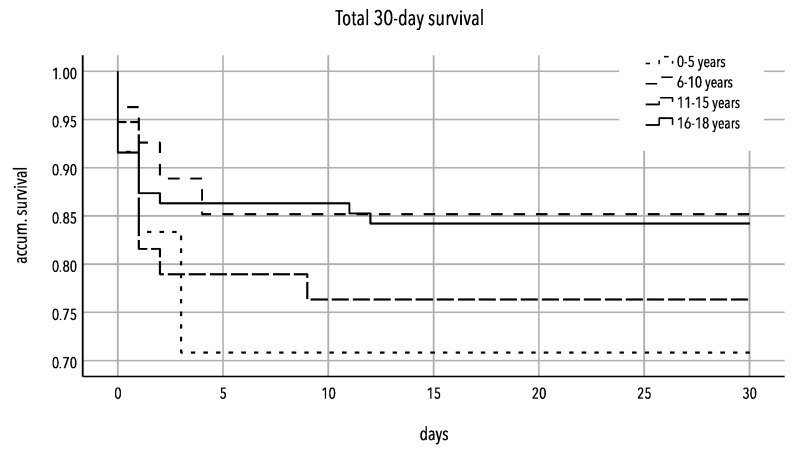
Kaplan–Meier curve of 30-day survival rate of severely injured patients (ISS ≥ 16) 0–5 years vs. 6–10 years vs. 11–15 years vs. 16–18 years.

In the survival curve (Figure 3), it is shown that patients in the cohort had the highest frequency of mortality within the first 5 days.

### 3.6. Influence of Injury Patterns on Mortality

**Figure 4:**
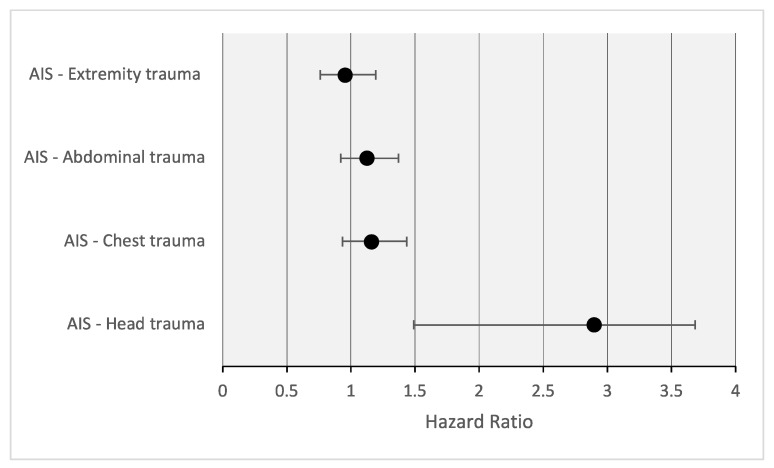
Correlation of injury patterns and mortality.

The univariate Cox regression analysis revealed a highly significant impact of head injury severity on mortality, with each additional AIS score point in the head region increasing the risk of death by 2.9 times (HR = 2.894 [1.489–3.687], *p* < 0.001), as shown in Figure 4. In contrast, injuries in other body regions did not exhibit a significant influence on mortality, although there was a trend towards an increased risk with severe injuries in the thoracic and abdominal regions (thorax: HR = 1.159 [0.935–1.434], *p* = 0.128; abdomen: HR = 1.123 [0.920–1.370], *p* = 0.254).

**Figure 5:**
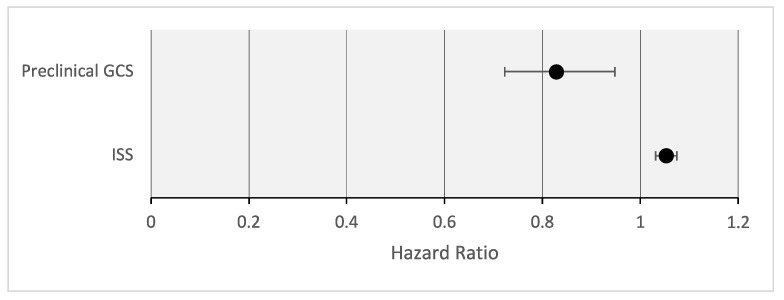
Multiple regression: valid predictors of mortality.

In the context of the multiple Cox regression analysis, the influence of the ISS (Injury Severity Score) and prehospital GCS (Glasgow Coma Scale) was confirmed. An increase in the ISS was significantly associated with an elevated risk of mortality (HR = 1.053 [1.031–1.075], *p* < 0.001), as shown in Figure 5. Similarly, a lower GCS score was significantly linked to an increased likelihood of mortality (HR = 0.828 [0.723–0.948], *p* = 0.006).

## 4. Discussion

In this prospective cohort study, we analyzed injury patterns, early clinical care, and outcomes in pediatric polytrauma patients. Two key findings emerged from this study: firstly, head injuries had the most significant impact on pediatric patient mortality, and secondly, the Injury Severity Score (ISS) and preclinical Glasgow Coma Scale (GCS) were identified as valid predictors of mortality in our patient collective. Age-related differences in injury severity and clinical interventions were minimal.

As observed in previous studies, boys were more frequently affected than girls [13,14,15]. The proportion of male patients significantly increased with age (*p* = 0.049). However, among patients under 10 years old, there was a nearly equal gender distribution. In a study by Søreide et al., which investigated deaths among children and adolescents due to severe traumatic injuries, a similar nearly equal gender distribution was found among children aged ≤13 years [16]. These findings suggest the emergence of more risk-prone behavior in males during adolescence. 

Regarding the preclinical and early clinical treatment procedures, several observations stand out. In this study, the percentage of intubated patients was 76.6%. Within the age group of 0–5 years, this percentage was even higher at 91.7%. This indicates a significantly higher rate of intubation in this study compared to other works in the literature [17]. In the study by Wyen et al., the intubation rate for toddlers aged 2–5 years was 50.9% [17]. The higher rate of intubated patients in our study can be attributed to the greater severity of injuries. Furthermore, a high rate of resuscitation was observed, particularly in the 0–5 years age group. Wyen et al. report similar findings in their study and pose two possible reasons for these differences between the groups [17]. On one hand, it can be explained by the occurrence of different injury patterns, with a significantly higher proportion of life-threatening traumatic brain injuries in this age group. On the other hand, making the decision to withhold or terminate resuscitation attempts in the field is challenging, especially when dealing with children, where emotions run high among all healthcare providers [18]. Due to these challenges, paramedics and emergency physicians might delay CPR until reaching the Emergency Department (ED) when dealing with pediatric cases. These assumptions can be further supported through our results, as none of the patients transported to the ED under resuscitation efforts survived. 

Furthermore, accidents in road traffic were primarily responsible for polytrauma in children and adolescents in our cohort. Similar results were observed when comparing with the international literature. According to these studies, approximately 24–41% of polytraumas in children and adolescents were caused by collisions with vehicles [19,20]. Debus et al. described that the mechanisms of accidents in road traffic varied with age, ranging from pedestrian accidents to bicycle or motorcycle accidents and car accidents, reflecting age-appropriate roles in road traffic, as they implicate that trauma mechanisms change with the range of action of children [1]. This is also reflected in the results of our study. When comparing the different age groups, it becomes apparent that in children under 5 years of age, various other injury patterns as well as falls from great heights were the leading causes of trauma. 

The injury patterns vary with the patient’s age, as evidenced in the results of this study. When examining injuries with an AIS ≥ 2, a distinct distribution of head, abdominal, and extremity injuries was observed. Children aged 0–5 years primarily had head injuries. In the group aged 11–15-years, the prevalence of these injuries was lowest at 60.5%. Conversely, as the age groups increased, the likelihood of severe extremity and abdominal injuries also rose. This vulnerability to injuries in these body regions can be attributed to the disproportionately large head and weaker neck muscles in children [17]. Even at low speeds, there is a high risk of head injuries, linked to the absence of age-appropriate child restraint systems and the child’s positioning in the center seat [21,22]. Furthermore, severe head injuries appear to be the key determinant of mortality in pediatric trauma [23,24,25,26]. This held true both for injuries to a single organ system and injuries involving multiple organ systems [26]. Our study showed similar results, as the maximum AIS of the head was the strongest predictor of mortality. In contrast to the results of other studies, injuries to other body regions in the context of our study did not show a significant influence on mortality. In the literature, thoracic injuries are discussed as a relevant predictor of severe injuries with increased mortality and increased complications [15,27]. Although thoracic injuries appear to be rare, they are linked to high overall mortality rates [14]. Since children’s ribs are more elastic, thoracic trauma in children can have significant consequences and lead to increased lung and mediastinal injuries [15]. The lack of a significant influence of thoracic injuries on mortality in contrast to the results of previous studies could be explained by the size of the cohort, as there was a clear trend regarding the impact of thoracic injuries on mortality.

The results of our study demonstrated that the trauma scoring systems used to assess polytraumatized children and adolescents are valid predictors of mortality. Both the Glasgow Coma Scale (GCS) and the Injury Severity Score (ISS) are suitable for identifying critically ill children. A lower preclinical GCS score was significantly associated with a higher likelihood of mortality. Similarly, an increasing ISS was significantly correlated with an elevated risk of mortality. The ISS remains the most widely used trauma score to date [28]. However, its application for evaluating injured pediatric patients has been debated due to the physiological and anatomical differences between adults and children [26]. Despite the availability of specific scoring systems for children, the ISS continues to be the standard for assessing pediatric trauma patients [28,29]. The selection of the Injury Severity Score (ISS) in our study was driven by its established use as a widely recognized and accepted metric for assessing trauma severity in pediatric patients. While the recent literature discusses alternative scoring systems to compensate for the deficiency in evaluating polytraumatized children [30,31], the ISS remains the most widely used tool for trauma evaluations, offering a comprehensive overview of injury severity [31]. However, it is essential to consider these scores as part of a comprehensive prognostic model and conduct further research to determine their clinical relevance and applicability to pediatric patients in order to improve the survival rates of these patients. In the context of our study, the potential differences in trauma management have to be pointed out, particularly in tertiary centers equipped with specialized pediatric care facilities, where the availability of subspecialists and advanced medical resources may contribute to nuanced and comprehensive approaches to pediatric trauma care. Considering the evolving landscape of pediatric trauma care, our findings underscore the importance of tailored management strategies in tertiary centers with dedicated pediatric resources. A recent study by Snyder et al. [32] provides a comprehensive framework, fostering a deeper understanding of resource utilization in pediatric trauma centers through a Delphi expert panel. Furthermore, the study by Gatto A et al. [33] emphasizes the importance of a pediatric observation unit in managing children admitted to the emergency department, providing practical insights that can inform strategies for enhancing pediatric trauma care. Integrating these findings into the broader context of tertiary care facilities with specialized resources allows for a comprehensive discussion on refining management approaches and advancing the quality of care for pediatric trauma patients.

### Limitations

The goal was to provide an overview of the effects of injury, imaging diagnostics, and patient survival. It is crucial to acknowledge that the results of this study are constrained by the nature of the analysis, imparting a descriptive character to our findings. The observational design limits our ability to establish causal relationships or draw inferences about the efficacy of specific interventions. Similarly, the detailed evaluation of image files and correct diagnosis of individual patients was not possible due to our approach. In the context of this study, we decided to use the age groups mentioned above. However, especially in the group of 0–5-year-olds, different developmental stages are grouped together, which means that no highly differentiated classification with regard to age is made. A noteworthy limitation of our study pertains to the substantial discrepancies in sample sizes among age groups, primarily influenced by the scarcity of cases in the <2-year age cohort. Acknowledging this challenge, we chose to maintain broader age categories to uphold statistical robustness, recognizing that these variations may impact the precision of specific subgroup analyses within the pediatric population.

The relatively high occurrence of accidents in agriculture or related to livestock, categorized under various other injury patterns in this study, may be explained by the location of our hospital. The hospital serves a large and predominantly rural catchment area, which must be mentioned when analyzing the results.

Furthermore, this was a monocentric study, which limits the generalizability of the results. While acknowledging the single-center design, this study’s external validity may be influenced by the specific trauma patterns and care practices at the institution. Further studies could explore potential diversity in pediatric trauma care across various centers, with particular attention to distinctions between rural and urban settings. The deliberate focus on the most severely injured patients presents another key limitation. This intentional approach, while providing insights into a specific subset, may not fully represent the entirety of pediatric trauma. Future investigations could benefit from encompassing a broader spectrum of injury severity. However, the results of our study provide a good basis for further studies in this area, including inter-institutional or national studies. Nevertheless, this study is based on a large regional collective and provides a sound insight into the care of polytraumatized children and adolescents in the shock room of our Level 1 trauma center. While this study offers valuable observational results, further randomized controlled trials are necessary to investigate the topic.

## 5. Conclusions

Pediatric polytrauma patients in this Level I trauma center exhibit diverse injury patterns and clinical characteristics. Although age-related differences in overall injury severity and clinical interventions were limited, head trauma emerged as a critical predictor of mortality. This study identified the Injury Severity Score (ISS) and preclinical Glasgow Coma Scale (GCS) as indicators of mortality risk, emphasizing the importance of early assessment and resuscitation. These observations call for nuanced approaches to pediatric polytrauma care, considering age and injury patterns, to optimize outcomes. However, the observational nature of this study warrants caution in making conclusive claims about intervention benefits, highlighting the need for further research, potentially through controlled trials, to refine clinical guidelines for pediatric polytrauma patients.

## Figures and Tables

**Table 1:** Demographic data of analyzed patients.

	Total	0–5 Years	6–10 Years	11–15 Years	16–18 Years
Number (n)	184	24	27	38	95
Age *	16 (9.25; 17)	3 (1; 4)	9 (6; 9)	14 (13; 15)	17 (16; 18)
Male (n/%)	119/64.7	12/50.0	14/51.9	23/60.5	70/73.7
ISS *	29 (22; 38)	29 (21.25; 40.25)	24 (21; 25)	29 (21; 38.75)	30 (22; 41)
Preclinical GCS *	9 (3; 14)	8 (4; 10)	9 (3; 14)	9 (4; 15)	10 (3; 14)
Intubated (n/%)	141/76.6	22/91.7	23/85.2	28/73.7	68/71.6
*Thereof preclinical* (n/%)	132/93.6	20/90.9	22/95.7	26/92.9	64/94.1
Thoracic drainage (n/%)	35/19.0	4/16.7	3/11.1	8/21.1	20/21.1
*Thereof bilateral* (n/%)	13/37.1	3/75.0	1/33.3	1/12.5	8/40.0
Preclinical CPR (n/%)	24/13.0	5/20.8	3/11.1	5/13.2	11/11.6
*Thereof successful until* *emergency room* (n/%)	19/79.2	3/60.0	2/66.7	5/100.0	9/82.8
30-days mortality (n/%)	35/19.0	7/29.2	4/14.8	9/23.7	15/15.8
Time of hospitalization *	14 (6; 21)	13 (3; 23)	13 (7; 18)	11 (2; 19)	14 (9; 21)

* median [25th; 75th percentile].

**Table 2:** Trauma mechanism in different age groups.

	Total	0–5 Years	6–10 Years	11–15 Years	16–18 Years
Car (n/%)	63/34.2	5/20.8	8/29.6	11/28.9	39/41.1
Motorcycle (n/%)	42/22.8	0	0	5/13.2	37/38.9
Bicycle (n/%)	14/7.6	0	5/18.5	7/18.4	2/2.1
Pedestrians (n/%)	18/9.8	4/16.7	6/22.2	2/5.3	6/6.3
Fall ≥ 3 m (n/%)	24/13.0	6/25.0	3/11.1	6/15.8	9/9.5
Fall ≤ 3 m (n/%)	6/3.3	2/8.3	2/7.4	1/2.6	1/1.1
Others (n/%)	17/9.2	7/29.2	3/11.1	6/15.8	1/1.1

**Table 3:** Distribution of injury patterns by ISS body region: head; chest; abdomen; and extremities.

	Total	0–5 Years	6–10 Years	11–15 Years	16–18 Years
AIS–Head trauma *	4 (2; 5)	4 (3.25; 5)	4 (3; 4)	3 (1; 4)	4 (1; 5)
AIS–Chest trauma *	3 (0; 3)	3 (0; 3.75)	2 (0; 3)	3 (0; 4)	3 (0; 3)
AIS–Abdominal trauma *	0 (0; 2)	0	0	0 (0; 3.25)	2 (0; 2)
AIS–Extremity trauma *	2 (0; 3)	0 (0; 2)	2 (0; 2)	2 (0; 3)	3 (0; 3)

* median (25th; 75th percentile).

**Table 4:** Injury patterns of severe injury AIS ≥ 2.

	Total	0–5 Years	6–10 Years	11–15 Years	16–18 Years
AIS–Head trauma (n/%)	140/76.1	23/95.8	25/92.6	23/60.5	69/72.6
AIS–Chest trauma (n/%)	129/70.1	16/66.7	17/63.0	26/68.4	70/73.7
AIS–Abdominal trauma (n/%)	78/42.4	4/16.7	6/22.2	18/47.4	50/52.6
AIS–Extremity trauma (n/%)	116/63.0	8/33.3	15/55.6	24/63.2	69/72.6

## Data Availability

The data presented in this study are available on request from the corresponding author. Access to the source dataset is only permitted to employees of the Department of Trauma Surgery, University Medical Centre Regensburg.

## Abbreviations

AIS	Abbreviated Injury Scale
CPR	Cardiopulmonary Resuscitation
ED	Emergency Department
GCS	Glasgow Coma Scale
ISS	Injury Severity Score
